# Supplementary material for: The regulatory domains of the lipid exporter ABCA1 form domain swapped latches
Source: PLoS One. 2022 Feb 4;17(2):e0262746. doi: 10.1371/journal.pone.0262746 (PMC8815970; doi:10.1371/journal.pone.0262746)
Supplement: S1 Fig — Sequences were obtained from the NCBI database according to the methods in the main text. The optimal tree is drawn to scale using the indicated ABC-A sequences with branch lengths in the same units as those of the evolutionary distances used to infer the tree. Distances are in the units of the number of amino acids substitutions per site. The percentage of replicate trees in which the associated taxa clustered together in the bootstrap test using 500 replicates (ref 34) and are shown at the branchpoints. The tree shows that ABCA4 and ABCA7 are closely related to ABCA1 sequences. (DOCX) [file pone.0262746.s001.docx]

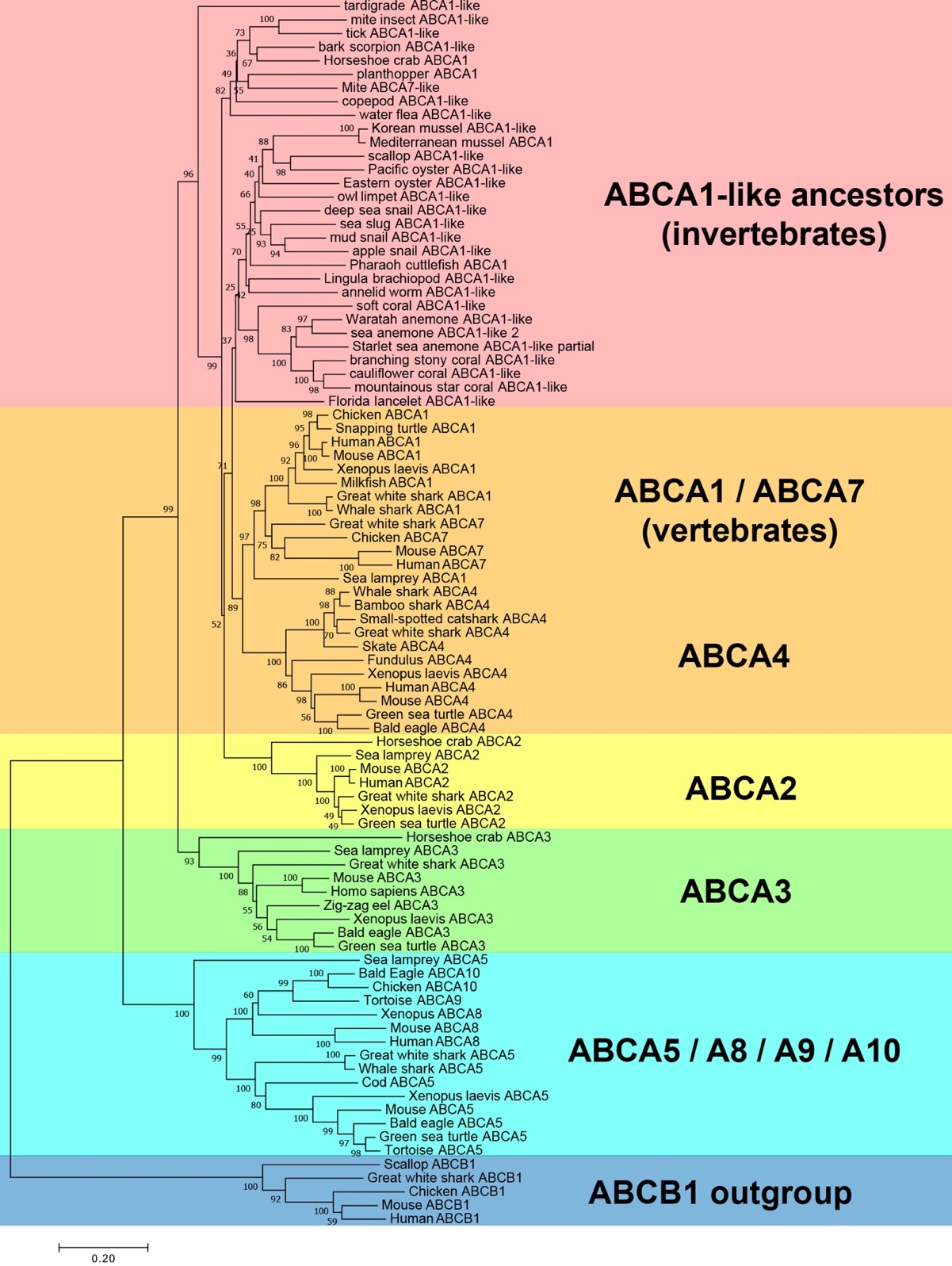


**S1 Fig. Phylogenetic relationships of the clades formed by the ABC-A subfamily.** Sequences were obtained from the NCBI database according to the methods in the main text. The optimal tree is drawn to scale using the indicated ABC-A sequences with branch lengths in the same units as those of the evolutionary distances used to infer the tree. Distances are in the units of the number of amino acids substitutions per site. The percentage of replicate trees in which the associated taxa clustered together in the bootstrap test using 500 replicates (ref 34) and are shown at the branchpoints. The tree shows that ABCA4 and ABCA7 are closely related to ABCA1 sequences.
