# Supplementary material for: The regulatory domains of the lipid exporter ABCA1 form domain swapped latches
Source: PLoS One. 2022 Feb 4;17(2):e0262746. doi: 10.1371/journal.pone.0262746 (PMC8815970; doi:10.1371/journal.pone.0262746)
Supplement: S2 Fig — The figure shows a compressed view of the entire alignment for all amino acid residues comprising the full transporter for ~80 distinct ABC-A individuals (from S1 Fig). Sequences for nucleotide binding and inner leaflet ligand binding were recognized from the high-resolution ABCA4 structure and are nearly invariantly conserved for the entire sub-family, as indicated by red color. “Cross over” and “Cross back” sequences are also fundamentally conserved for the entire sub-family. (DOCX) [file pone.0262746.s002.docx]

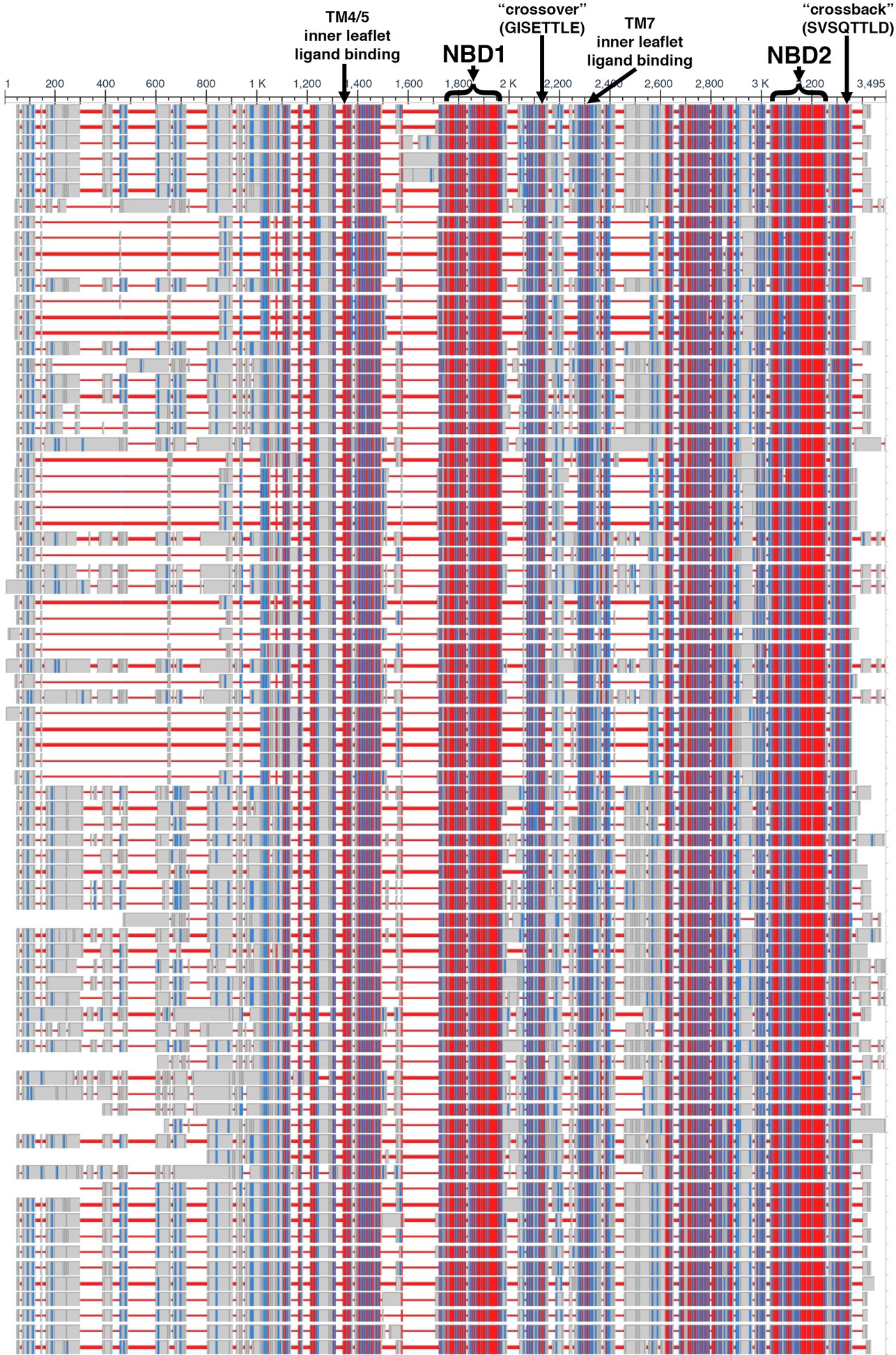


**S2 Fig. Conservation of amino acid residues involved in fundamental transport functions for the ABC-A subfamily, including invertebrates.** The figure shows a compressed view of the entire alignment for all amino acid residues comprising the full transporter for ~80 distinct ABC-A individuals (from Fig. Supp. 1). Sequences for nucleotide binding and inner leaflet ligand binding were recognized from the high-resolution ABCA4 structure and are nearly invariantly conserved for the entire sub-family, as indicated by red color. “Cross over” and “Cross back” sequences are also fundamentally conserved for the entire sub-family.
