# Supplementary material for: The regulatory domains of the lipid exporter ABCA1 form domain swapped latches
Source: PLoS One. 2022 Feb 4;17(2):e0262746. doi: 10.1371/journal.pone.0262746 (PMC8815970; doi:10.1371/journal.pone.0262746)
Supplement: S3 Fig — Panel A: partial cholesterol ligand was modeled in ABCA1 (our structure) near residues (Leu745, Phe1347, Leu1353, Phe1667, Tyr1767 and Val1768) as supported by original electron density. Panel B: full cholesterol molecule, as modeled by the original authors, in the corresponding site of ABCA4 (Leu760, Lys1371, Leu1379, Phe1692, Tyr1792 and Val1793). Panel C: partial cholesterol ligand was model in ABCA1 (our structure) near residues Thr1765, Val1769 and Ser1772 supported by original electron density. Panel D: partial cholesterol molecule, as modeled by the original authors, in the corresponding site of ABCA4 near residues Thr1790, Val1793 and Cys1797. (DOCX) [file pone.0262746.s003.docx]

**
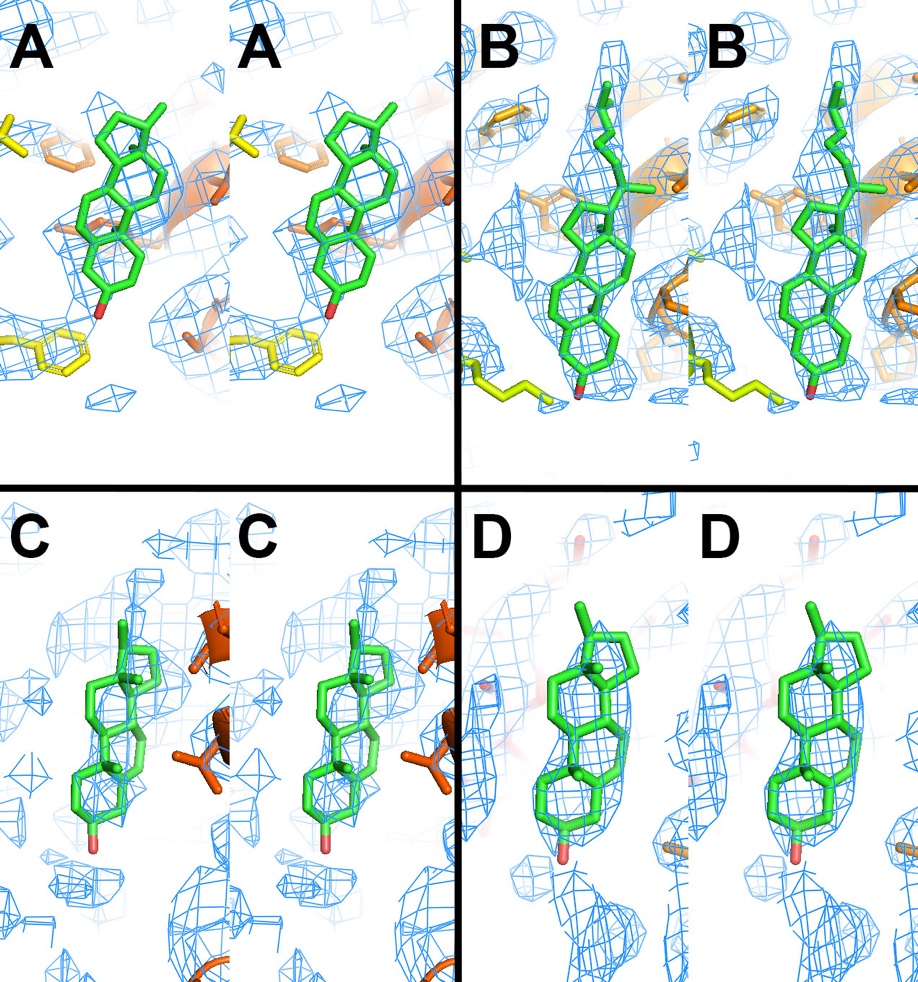
**

**S3 Fig. Conservation of ligand binding in the inner leaflet of ABCA1 and ABCA4. Panel A:** partial cholesterol ligand was modeled in ABCA1 (our structure) near residues (Leu745, Phe1347, Leu1353, Phe1667, Tyr1767 and Val1768) as supported by original electron density. **Panel B:** full cholesterol molecule, as modeled by the original authors, in the corresponding site of ABCA4 (Leu760, Lys1371, Leu1379, Phe1692, Tyr1792 and Val1793). **Panel C:** partial cholesterol ligand was model in ABCA1 (our structure) near residues Thr1765, Val1769 and Ser1772 supported by original electron density. **Panel D:** partial cholesterol molecule, as modeled by the original authors, in the corresponding site of ABCA4 near residues Thr1790, Val1793 and Cys1797.
