# Supplementary material for: The regulatory domains of the lipid exporter ABCA1 form domain swapped latches
Source: PLoS One. 2022 Feb 4;17(2):e0262746. doi: 10.1371/journal.pone.0262746 (PMC8815970; doi:10.1371/journal.pone.0262746)
Supplement: S1 Table — Alpha-carbons with data type as helix (i.e. “H”) or sheet (i.e. “S”) were taken directly from their secondary structure assignment within PyMOL. The average percentage ± SD of β-sheet for all 11 eukaryotic transporters was 7.8 ± 1.5%. (DOCX) [file pone.0262746.s004.docx]

**S1 Table. Secondary structure calculation of 31 different ABC transporters measured directly from the indicated PDB depositions.** Alpha-carbons with data type as helix (i.e. “H”) or sheet (i.e. “S”) were taken directly from their secondary structure assignment within PyMOL. The average percentage ± SD of β-sheet for all 11 eukaryotic transporters was 7.8 ± 1.5 %.
