# Supplementary material for: The regulatory domains of the lipid exporter ABCA1 form domain swapped latches
Source: PLoS One. 2022 Feb 4;17(2):e0262746. doi: 10.1371/journal.pone.0262746 (PMC8815970; doi:10.1371/journal.pone.0262746)
Supplement: Attachment — Submitted filename: PONE-D-21-34405-Response_to_PCOMPBIOL-D-21-01625.docx [file pone.0262746.s005.docx]

**Note to Editors (PONE-D-21-34405): The following point-by-point responses have already been incorporated into the Revised version that has been uploaded to the PONE website.**

Segrest et Aller, manuscript #PCOMPBIOL-D-21-01625

Comments in black font were copied verbatim from the Reviewer’s comments and questions.

Author responses are colored blue below.

**PCOMPBIOL-D-21-01625 Reviewer #1**

We wish to thank the reviewer for the very positive comments regarding our manuscript, in particular for the suggestion that “the manuscript be accepted” after addressing the comments. We provide a point-by-point response including our actions taken to revise our manuscript accordingly:

Aller and Segrest presented a dispute on the correction position of the regulatory domains of the lipid exporter ABCA1 in their manuscript “The Regulatory Domains of the Lipid Exporter ABCA1 Form Domain Swapped Latches.” In the manuscript, the authors performed rigorous sequence alignment analyses across all ABCA subfamily members and built a new structural model for ABCA1. Overall, the claim is well supported by the existing evidence, and it is extremely important for the researchers who work on characterizing ABCA1 functions to have a more accurate model out there. I would suggest the manuscript being (sic) accepted after the following comments are addressed.

1. on line 127, for figure 4, you mentioned “several amino acid residues in that region are completely invariant”. For clarity for the readers, could you label the invariant ones with specific label on the figure (such as “*”)? *** This is a very good suggestion for clarifying Figure 4. There are five invariant residues in the “crossover” and five invariant residues in the “crossback”. We have labeled these 10 positions with an asterisk (i.e. *) in our Revised manuscript and we have indicated so in the revised legend for Figure 4.

2. from line 173-175, you mentioned about cholesterol molecule densities for ABCA1 and ABCA4 and proposed on line 179 “...raises tantalizing possibility that one or more of the molecules are poised to be flopped onto the outer leaflet…” Could it be a bit overinterpreting? The density in the inner leaflet of ABCA1 is not clear enough that they are cholesterol molecules. According to Qian et al.’s paper on the ABCA1 structure, they purified proteins in DDM and CHS detergent. Putting in another way, the proteins are not present in their natural environment for such hypothesis to be sounding. *** The Reviewer raises an interesting point. As we pointed out in the manuscript, the possibility of the putative cholesterol binding site was also raised by the original 2017 publishing authors (Qian et al), although those authors were not confident to model any atoms in their original model even though their own CryoEM density supports the presence of cholesterol or a cholesterol-like ligand (i.e. CHS). Five years after the ABCA1 structure was published, Dr. Jue Chen’s group published two structures of ABCA4, one of which is nucleotide free (as with ABCA1). Nucleotide-free ABCA4, solved to 3.4 Å resolution, exhibits a clear cholesterol binding site that overlaps with the ABCA1 site when the two protein structures are aligned. We also point out that cholesterol was modeled even though the ABCA4 protein was purified in the presence of CHS (see PDB entry 7LKP). Furthermore, the common cholesterol binding site between ABCA1 and ABCA4 is formed by highly conserved amino acid residues. Since CHS and cholesterol have the same atoms comprising their steroid-like moiety, we propose it is appropriate to model the cholesterol in our updated model. However, we have softened the language in our revised manuscript to consider the possibility that the ligand is either cholesterol or CHS.

3.  line 183 to 185, “The implications for the subfamily’s transport mechanism are significant and suggest that ABCA transporters can process substrate because their NBDs stay in constant proximity”. Is there specific evidence for it? The mechanism of ABCA family transport is quite unclear now. If there are evidence that deletion of R domain is necessary for the transport, please cite the papers to support your claims. *** We thank the Reviewer for this point. Our conclusion paragraph has been re-written to clarify two conclusions and one speculation. The two conclusions are: 1) we produce a more accurate model of ABCA1, based on original CryoEM density, that contains the regulatory domains modeled as domain swapped and 2) the domain swapping is conserved in the entire ABC-A subfamily. The speculation is that the domain swapping, which is common between an exporter (ABCA1) and a known importer (ABCA4) directly imply that the ABC-A subfamily may use a mechanism quite distinct from canonical ‘alternating-access’.

4. In your uploaded PDB structure, a bit portion of building is missing in NBD2. I understand that the density might not be good enough for building. However, given NBDs are quite conserved, is it possible for you to model in that part for the general interest of the public based on the known ABCA4 structures? *** Yes, it is highly possible, and we were very tempted to do so. However, our close inspection of the original CryoEM density surrounding this region is weaker than the rest of the NBD, and we chose to be conservative and remove the modeled atoms until they are supported better by future data.

5. Alphafold database has released the predicted structure of ABCA1 and it is indeed domain swapped on the R domains. It would be nice if you could add an extra figure doing the comparison or use that to support your hypothesis. *** Indeed, we were aware of the Alphafold prediction of ABCA1 during the preparation of the manuscript, and we agree with the Reviewer that it would be appropriate for us to include a statement and we have added one to the manuscript.

**PCOMPBIOL-D-21-01625 Reviewer #1**

Reviewer #2: The authors report the re-refinement of a recently published cryo-EM structure of the ABCA1 lipid exporter, correcting a consequential modelling error in a regulatory domain that places ABCA1 back into the group of canonical ABC transporter structures, including the ABCA4 importer.  The authors support their re-analysis of the electrostatic potential maps with a phylogenetic analysis that shows that ABCA1 has the conserved “crossover” region shared by other ABC transporters that feature a domain-swapped regulatory domain.  Interpreting electrostatic potential maps at >3 Å resolution can be challenging, particularly when domain swapping is involved, and the authors’ combined bioinformatic and structural approach takes an appropriately parsimonious approach.  In all, the analysis is convincing and the paper is clearly written.  Having said this, the manuscript does not report new data or methods and the re-analysis, though thoughtful, does not break new computational ground.  I expect that this paper will be of interest to researchers working on the structures of ABC transporters.  I include detailed comments below. ***We wish to thank the reviewer for the very positive comments, particularly for stating that our “analysis is convincing and the paper is clearly written”. We provide a response to the major- and minor criticisms here:

Major issues:

1)	I appreciate the central (and too-often neglected) importance of improving the literature by correcting prior misinterpretations. I commend the authors for taking on this task and making a convincing argument.  However, the drawback of doing such work is that it often is of intense but narrow interest to researchers working on that particular system, which I suspect may be the case here. ***We disagree that our findings are of narrow interest. We show that 1) the exporter ABCA1 contains a domain swap as with the importer ABCA4 and 2) we show the domain swap architecture is conserved throughout the ABC-A sub-family of ABC transporters. The implications on the mechanism of transport are potentially substantial, and our new ABCA1 model will allow many competent groups the opportunity to probe the dynamics of transport through computational studies, such as all-atom MD.

Minor issues:

2)	Technically, the density map resulting from cryo-EM is an electrostatic potential (or Coulomb potential or electron potential) map rather than an electron density map, as the authors state.  The complication arises because the negative charge of the electron introduces an electrostatic consideration into the scattering factors that is not present for X-rays, which scatter from electron density. ***We thank the Reviewer for this important point, and we realize that we were careless with our terminology. In fact, this seems to have been a problem in the literature for many years. Please see the following (among others) that use the term “electron density” when referring to CryoEM density maps specifically:

Fleishman, Harrington, Enosh, Halperin, Tate & Ben-Tal *J.Mol.Biol.* 364, 54-67 (2006)

Adrian, Dubochet, Fuller & Harris, *Micron* Vol 29, pp145-160 (1998)

Russell et al, *Curr. Op. Struc. Biol.*, 14:313-324 (2004)

O’Donoghue et al, *Nature Methods*, 7:3 (2010)

Costa et al, *Nature Struc. & Mol. Biol.* 18(4):471-7 (2011)

Meusch et al, *Nature* 508:61-ff (2014)

In order to be more accurate, we have made changes throughout our revised manuscript to “CryoEM density”.

3)	Figure 5 gets the point across but is somewhat hard to parse.  In particular, the question mark label is not clear. *** We apologize for the difficulty with the figure. We noticed that one question mark (i.e. “?”) was missing in panel D, making that panel difficult to see. We have corrected this problem, and we have also changed the “crossover” label in panel B to “crossback” to distinguish from the first crossover.
