# Supplementary material for: The regulatory domains of the lipid exporter ABCA1 form domain swapped latches
Source: PLoS One. 2022 Feb 4;17(2):e0262746. doi: 10.1371/journal.pone.0262746 (PMC8815970; doi:10.1371/journal.pone.0262746)
Supplement: Attachment — Submitted filename: comments.docx [file pone.0262746.s006.docx]

The current manuscript describes the refinement and model correction of the previously published structure of the non-swapped regulatory domain of the ABC1 exporter. The study, supported by phylogenetic analyses and insights from the Alpha fold database, reports that the regulatory domain should be in swapped arrangements. This is a revised manuscript, and the authors have answered the comments and concerns raised by reviewers. Overall the manuscript is very well written, and the analysis is thoughtful and convincing. I don’t have further comments except for two minor typological errors given below. I recommend that now the manuscript can be formally accepted.

Minor comments.

- Page 4, line 98: There is a small typo error, “lathes” should be written as “latches”
- Minor comments: At some places, authors wrote “subfamilies” and “sub-families.” It would be better to keep it consistent throughout the manuscript.
